# Cost–benefit considerations have limited effect on the decision to exert cognitive effort in real-world computer-programming tasks

**DOI:** 10.1098/rsos.211885

**Published:** 2022-06-22

**Authors:** Itamar Lachman, Irit Hadar, Uri Hertz

**Affiliations:** ^1^ Department of Information Systems, University of Haifa, Haifa, Israel; ^2^ Department of Cognitive Sciences, University of Haifa, Haifa 3498838, Israel

**Keywords:** cognitive effort, decision-making, subjective value, programming, code reuse

## Abstract

Recent research shows that people usually try to avoid exerting cognitive effort yet they are willing to exert effort to gain rewards. This cost–benefit framework provides predictions for behaviour outside the laboratory. Nevertheless, the extent to which such considerations affect real-life decisions is not clear. Here we experimentally examined computer-programmers' decisions to write code in a reusable manner, using functions, which demands an initial investment of cognitive effort or to clone and tweak code, a strategy whose cost increases with repetitions. Only a small portion of our participants demonstrated sensitivity to the benefits and costs of programming strategies. When asked to solve the tasks, participants tended to avoid using functions, demonstrating biased effort estimation. By contrast, when asked how they *planned* to solve the tasks, participants tended to demonstrate an opposite trend, overestimating effort, in line with an injunctive norm involving the overuse of functions. In the context of real-world problems, the effect of cost–benefit considerations may therefore be limited by task-irrelevant factors. Our interdisciplinary approach may be useful in providing novel theoretical insights and in promoting cognitive-effort investments outside the laboratory.

## 1. Introduction

In recent years, our understanding of how people decide whether to exert cognitive effort has greatly improved, providing a new theoretical framework for understanding and characterizing cognitive effort allocation [1–3]. This framework is based upon the observation that, as in the case of physical effort and pain, people try to avoid exerting cognitive effort [4,5], yet are willing to engage in tasks that require cognitive effort to obtain rewards [6–8]. Inspired by economic models of bundled goods, this framework highlights the role of cost–benefit considerations in people's decisions regarding how much cognitive effort to exert. This notion has been demonstrated in a variety of cognitive tasks in the laboratory that involve working memory, attention, executive control and planning [6–10]. This framework can be extended to other tasks beyond the laboratory to predict decisions in a variety of real-life contexts. However, it is not clear how these cost–benefit considerations fare in real-life tasks, especially when other contextual factors may also play a role. Here we consider a problem that professional and casual programmers face every day: deciding which computer-programming strategy to use. Cognitive effort estimation is poised to have a considerable effect on such decisions.

Imagine that upon completing data collection from participants you discover a lag in the measurements, such that all measures from a particular participant must be updated before the data are ready for further analyses. Luckily, this can be easily solved by means of a computer-programming script that loops through the measurements and updates them. But what if more than one participant needs to be updated and that each has a different lag? One strategy is to clone the script and tweak it to suit each participant. Another strategy is to write a piece of reusable code, that is, a function that can be deployed in many contexts with different parameters. Writing reusable code demands more cognitive effort, as the programmer needs to consider a more abstract and general version of the problem [11–16]. In the long run, however, writing reusable code is beneficial because it reduces the time and effort involved in developing and maintaining the code in the future [17–19]. Indeed, managers and employers [20] as well as many guides and textbooks recommend writing reusable code as the preferred strategy whenever an operation needs to be used more than once [17,21,22].

This problem lends itself to examining the cost–benefit framework in deciding whether to exert cognitive effort. The cognitive effort associated with the clone-and-tweak method is dependent on the number of repetitions in the task, i.e. how many cloning steps are needed. The strategy of code reuse demands a one-time allocation of cognitive effort that is not proportional with the number of repetitions. Thus, cost–benefit considerations should compare the cognitive effort associated with each strategy to determine the most efficacious one, i.e. lowest effort for best outcome (also termed *control efficacy* [1]) [23]. Such a decision should, in principle, be sensitive to the number of repetitions in a task: when the number of repetitions is low, the accumulated effort associated with clone-and-tweaks will probably not exceed the effort associated with writing a function (a type of reusable code), such that the likelihood of writing a function should increase with the number of repetitions. Thus it seems that the cost–benefit framework of cognitive effort can provide predictions for the decisions made in computer programming.

Other factors, however, may affect this decision. These factors may predict other patterns of decisions that are not sensitive to the number of task repetition of the tasks and hence do not represent cost–benefit considerations. For example, overestimating the initial investment of cognitive effort may bias code writers [24,25]. When an option demands a large initial investment, the programmer is liable not to plan beyond the initial cost and is likely to disregard future benefits that may offset this investment [26]. As planning is in itself an effortful, and therefore costly, task [5,27], a programmer may focus only on the initial costs of each strategy and exhibit a rigid pattern of function avoidance that is insensitive to task demands. This avoidance of code reuse has been observed in pupils and professional computer programmers [28,29]. This pattern does not contradict the cost–benefit framework *per se*, as the input to the cost–benefit function, i.e. the effort estimation, is biased. Yet it differs from the more general version given above as it predicts a rigid pattern of decisions that is less sensitive to context.

Code writers may also follow injunctive norms—i.e. rules that describe what people should do in a particular situation—rather than considering the task at hand [30–32]. In many cases, people are willing to exert extra effort to follow an injunctive norm, for example by waiting for the pedestrian traffic signal to turn green even when no cars or other people around. Indeed, people can differentiate between injunctive norms, e.g. whether people should drink and drive, and descriptive norms that describe what most people actually do, e.g. how likely are people to drink and drive [33,34]. As already mentioned, in computer programming the strategy of code reuse and function writing is hailed as best practice [17,21,22]. A programmer following such a strategy may therefore be less sensitive to the number of repetitions in a task and thus exert the additional cognitive effort of writing functions even when the number of repetitions is very low. The effect of cost–benefit considerations in making real-life decisions about whether to exert cognitive effort may be confounded by such injunctive behavioural prescriptions [34].

In this study, we set out to examine how cost–benefit considerations affect programmers' decisions to write reusable code. We conducted three experiments in which we manipulated the number of repetitions within a coding task. All participants were students majoring in information systems. They were asked to do one of the following: (1) solve a task; (2) declare how they intend to solve a task (to overcome initial cognitive effort estimation); (3) plan and then solve a task. By examining the overall likelihood that an individual will solve a task by writing reusable code and the relationship between this likelihood and the number of repetitions in the task, we can provide evidence for the contribution of cost–benefit considerations in such real-world problems. Our main measure is the relationship between the number of repetitions in the task and the likelihood of function writing, and how it changes across experimental conditions, e.g. gradually increasing relationship or nonlinear one. We cautiously examine the time it took participants to complete each task, as one benefit of function use is that it allows faster solutions to highly repetitive tasks. Note that in addition to providing theoretical insights and predictions for programmers, our approach can inform real-life promotion of cognitive effort investments.

## 2. Material and methods

### 2.1. Participants

Our participants were 148 students in the Department of Information Systems at the University of Haifa. We aimed at a sample size of 40 participants per experiment, as we followed a similar within-subject design similar to Westbrook *et al*. 2013 [7]. We evaluated our sample size power when comparing distributions of function use between experiments (coding in Experiment 1 compared with experiment 3 resulted in effect size of 1.69, and power of 0.9 with our sample size) and within experiment (eta-square of repetition effect in Experiment 1 was 0.13, with power greater than 0.9 with our sample size and within-participants design). The participants in Experiments 1 and 3 were first-year students who had completed two basic programming courses (C and Java). The participants in Experiment 2 were third-year students who had completed additional and related advanced courses (Software Design and Software Engineering). Nine participants were excluded from the analysis as they did not meet our screening criteria (see §2.3 Data preparation). After this exclusion, we analysed data from 139 participants: Experiment 1 (*N* = 41, 18 female, 23 male [*M*(age) = 22.1, s.d. = 2.62, range = 19 to 29]; Experiment 2 (*N* = 50, 22 female, 28 male [*M*(age) = 25, s.d. = 2.6, range = 21 to 31]; Experiment 3 (*N* = 50, 26 female, 24 male [*M*(age) = 21.9, s.d. = 2.3, range = 18 to 27].

The experiments were approved by the Institutional Ethics Committee at the University of Haifa (Number 396/20), and each participant signed a letter expressing informed consent to participate in this study and confirming that all experiments were performed in accordance with relevant guidelines and regulations. Participants were compensated with course credit for their participation. Instead of participating in the experiment, students could choose an alternative non-research-related task and receive the same credit. The research team was not associated with the course.

### 2.2. Experimental design

**Figure 1.**
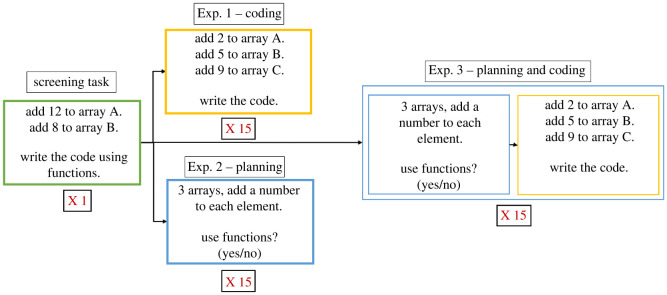
Design of the three experiments: Exp. 1 – coding, Exp. 2 – planning, and Exp. 3 – planning and coding. All experiments began with a compulsory screening task to confirm participant's ability to write functions. Those who did not meet this requirement were excluded from the dataset.

We conducted a series of three experiments, with the results of each experiment guiding the design of the subsequent one. Each experiment was conducted in a computer laboratory classroom via a dedicated web application. As a first step in the experiment, participants were required to complete a screening coding task (figure 1) in which they were asked to write a computer-programming script that adds numbers to elements of three arrays using a function. This task was aimed at verifying that the participants knew how to write a function. Participants who failed to complete this task correctly were excluded from the analysis. After this screening task, participants were asked to perform the main task. Participants were not instructed about function use beyond this point.

In our experiments, participants were given a series of simple computer-programming tasks (figure 1). In each task, they were asked to perform a mathematical operation on all the elements in an array by adding, subtracting, or multiplying by a given number. The number of arrays to be changed differed in each task, while the operation to be performed for each array remained constant within the task. This increased the number of repetitions per task, namely, the number of times the same operation was performed. For example, in a no-repetition task, participants were asked to add the number 8 to all the elements in array A, while in a five-repetition task participants were asked to add a different number to all the elements in each of the five arrays: A, B, C, D and E. Overall, participants were asked to perform 15 tasks in random order. The tasks entailed three operations (add, subtract, multiply) and five levels of repetition (1, 3, 5, 8 or 15 repetitions). According to cost–benefit considerations, an increase in the number of repetitions in a task should result in a greater likelihood that participants will write reusable code (functions) in their task solution, in our case manifested as a function.

Experiment 1 entailed coding tasks. A detailed task description (names of arrays and numbers to be added to/subtracted from/multiplied by each element) was displayed on the left-hand side of the screen, and a black window for writing the code appeared on the right-hand side of the screen. Participants could use copy and paste only within a task, as the clipboard was cleared between tasks.

Experiment 2 was designed to examine the effect of planning on the decision to use functions. It, therefore, included planning tasks. Participants were given a more abstract description of the task, which included the number of arrays to be manipulated and the operation to be performed on the arrays. Participants were asked to indicate whether or not they planned to use functions to solve this task. They were also told that after completing all the planning tasks, they would be required to code two of the tasks (to be randomly selected) according to their selected strategy during planning. We added this stipulation to induce participants’ commitment to their proposed plan.

Experiment 3 was designed to examine whether the observed planning effect translates to writing reusable code (i.e. using functions) and therefore included both planning and coding tasks. Each task was initially presented as a planning task, identical to the presentation in Experiment 2. Immediately thereafter participants were given a matching coding task, identical to the one presented in Experiment 1.

A detailed description of the instructions given to the participants is included in the electronic supplementary materials.

### 2.3. Data preparation

The data preparation included two steps: (i) exclusion of data from participants who failed to complete the preliminary task; and (ii) labelling each code solution written by participants as reusable or not.

Two experienced programmers external to the research served as evaluators in labelling the participants' code solutions. To be labelled as reusable, a code solution had to consist of a fragment of code that is surrounded by a single block and that can be used at another location. We also required that both evaluators evaluate the code as meeting the above criteria for it to be conclusively labelled as reusable. The inter-rater agreement between the evaluators was high (90%). Code solutions for which the evaluators did not agree were marked as solutions that were not reusable. A sensitivity analysis on this classification showed that the overall effect is not altered when considering all disagreements as reusable solutions (electronic supplementary material, table ST3). Input from the evaluators was also used to screen the participants based on their code in the screening task. A total of nine participants failed to complete the preliminary task in our experimental design and were excluded: one from Experiment 1, seven from Experiment 2 and one from Experiment 3.

### 2.4. Data analysis

We used mixed-effects logistic regression models to examine function use in coding and planning. Participants’ decisions (function/no function during coding/planning) served as the dependent variable, and the order of the task in the experiment and number repetitions served as dependent variables of interest. These multi-level models [35,36] include group-level coefficients, i.e. fixed effects and individual-level intercept coefficients of no interest, i.e. random effects. All models were fitted using the lme4 package in R [37].

Repetition levels were modelled in two different ways using two independent models. In the first model, repetition levels were modelled using an ordered scale from 1 (1 repetition) to 5 (15 repetitions). In the second model, repetitions were modelled using a two-level factor—either one repetition or more than one repetition—to capture the recommended practice heuristic used by the participants. The Bayesian information criterion (BIC) scores of the models were compared to identify the model that best fit the data.

## 3. Results

### 3.1. Experiment 1

Experiment 1 was designed to examine cost–benefit considerations in participant-programmers' decisions to write reusable code. In this experiment, participants were presented with multiple computer-programming tasks that included a varied number of repetitions (arrays to update). For each task, the participant was provided with the array names, operations and numbers to be used to update the elements in each array and then asked to write a computer-programming script to solve the task.

**Figure 2.**
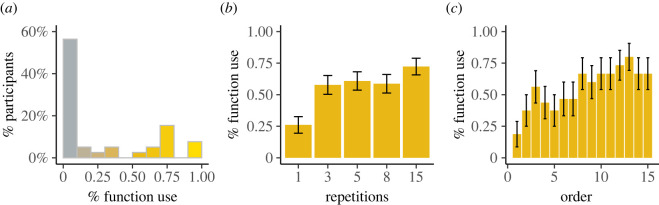
Programming decisions in Experiment 1. (*a*) Percentage distribution of programming with functions in Experiment 1. Most participants did not write functions at all and followed a clone-and-tweak strategy. The subset of participants who wrote functions at least once exhibited an increased likelihood to write functions when the number of repetitions increased (*b*), as well as later in the experimental order (*c*), in line with cost–benefit predictions.

In Experiment 1, we observed that most participants (22 out of 39) did not write a single function. Rather, they opted for a clone-and-tweak strategy, even when the same operation was performed for 15 different arrays (figure 2*a*). This pattern could arise from biased cost–benefit estimation in which the participants focused only on the immediate cognitive effort of code-reuse and clone-and-tweak strategies for one repetition and ignoring future costs and benefits. This pattern may also arise from indifference between the clone-and-tweak and reusable-code strategies, as participants may find it just as easy to copy and paste their solution, and write a function and calling it multiple times.

We then focused on the subset of participants who chose to write functions at least once (16 out of 39 participants) to better characterize the parameters underlying the decisions to use functions. We conducted a mixed-effect logistic regression on the participants’ decisions, where the decisions (1 = function, 0 = no function) were the dependent variable (the results from a model including all participants is available in electronic supplementary material, table ST1). The model included a group-level coefficient for the number of repetitions within each task (1,3,5,8,15) and the task order (1–15), i.e. the order of the task within the experiment. The results (table 1) show that the subset of participants in Experiment 1 who wrote functions at least once was affected by the number of repetitions (figure 2*b*) and by the task order (figure 2*c*). In other words, their tendency to write functions increased as the number of repetitions within a task increased, in line with the cost–benefit framework prediction. In addition, they were more likely to write functions in tasks given later in the experiment than in tasks given earlier. This effect may further indicate how people update their estimation after experiencing the effort associated with function avoidance, i.e. learning the marginal value of mental effort [38], supporting the biased effort-estimation account of avoiding the use of functions.

**Table 1.** Mixed-effect logistic regression analysis of the effect of within-task repetitions and task order on writing functions and planning to use functions. The models with the best fit are shown for each type of decision and experiment: the gradual repetition level model for Experiment 1 and the more-than-once (greater than 1) model for Experiments 2 and 3. (Full tables of alternative models are included in table ST2 in the electronic supplementary material ).

	estimate	s.e.	*z*	*p*
Experiment 1 – writing functions				
intercept	−3.64	0.76	−4.75	<0.001
repetition (gradual)	0.69	0.14	4.93	<0.001
order	0.23	0.046	5.03	<0.001
Experiment 2 – planning to use functions				
intercept	−1.79	0.39	−4.58	<0.001
repetition (>1)	4.19	0.34	12.23	<0.001
order	0.012	0.03	0.40	0.68
Experiment 3 – planning to use functions				
intercept	−1.64	0.41	−4	<0.001
repetition (>1)	4.95	0.40	12.27	<0.001
order	−0.05	0.03	−1.59	0.11
Experiment 3 – writing functions				
intercept	−3.64	0.59	−6.17	<0.001
repetition (>1)	5.28	0.52	10.2	<0.001
order	0.026	0.031	0.84	0.4

### 3.2. Experiment 2

Experiment 2 was designed to examine whether planning can overcome the tendency to avoid writing reusable code, which may stem from only taking into account the immediate cognitive effort required by the two programming strategies, or from indifference between the two coding strategies. In this experiment, participants were given a more abstract description of the task, including the number of arrays to be updated and the operation to be performed on each element in the arrays (figure 1). They were not asked to solve the task immediately but rather to indicate whether they planned to solve the task using functions. They were also told they would be required to implement two of the planning tasks (chosen randomly).

**Figure 3.**
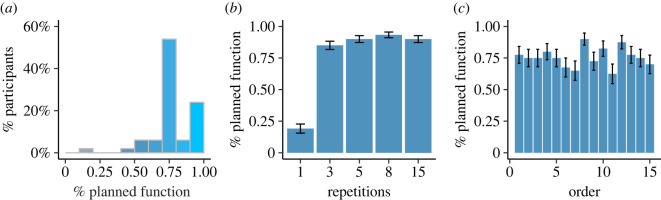
Programming decisions in Experiment 2. (*a*) Distribution of the percentage of participants who planned to code using functions in Experiment 2. Most participants stated they planned to use functions most of the time. The subset of participants who avoided functions at least once followed a more-than-once rule when planning to use functions (*b*), and did not show an order effect (*c*), exhibiting a pattern that was not sensitive to cost–benefit considerations.

In this experiment, all the participants indicated at least once that they would use functions to solve the task. Almost half (24 out of 50) planned to use a function in all the tasks that entailed more than one array, i.e. 12 out of the 15 tasks (figure 3*a*). This distribution differed significantly from the distribution observed in Experiment 1 (Kolmogorov–Smirnov test, *D* = 0.68, *p* < 0.001). This significant difference between planning and coding indicated that participants approached the planning and coding decisions differently. In both cases, the majority of the participants displayed a decision pattern that was not sensitive to the number of repetitions in the task. However, this insensitivity was manifested in different biases—away from functions in Experiment 1, and toward functions in Experiment 2.

To investigate participants' sensitivity to the number of repetitions in a task, we compared two logistic regression models. The first model was identical to the one used in Experiment 1, with the repetition effect coded as a gradual increase (1,3,5,8,15). To examine whether participants used a non-gradual strategy (*if more than one repetition then use a function*) that represented the injunctive norm in their decision to write functions, we fitted a second model to all decisions in which the repetition levels were conceptualized in terms of two levels only: ‘1’ for one repetition and ‘greater than 1’ for all other levels. We compared how well each of these two models fitted the data by examining their goodness of fit scores (Bayesian information criteria—BIC). Another account is a nonlinear increase in the cost of clone-and-tweak strategy with the number of repetitions. While we formally evaluated this model (electronic supplementary material, table ST4), this model did not perform as well as the other model, and is in stark contrast with the cost considerations observed in Experiment 1.

We fitted both models to the subset of participants who chose to avoid functions at least once (40 out of 50 participants). We found that decisions that entailed planning to write functions were markedly better modelled by the heuristics model (BIC gradual model: 513.88, BIC more-than-once model: 439.96, *χ*^2^ = 73.92). This indicates that the likelihood of planning to use functions did not gradually increase with the number of repetitions, but rather followed the *more than one repetition* injunctive norm, increasing from a very low likelihood to an almost fixed point when tasks included more than one repetition (figure 3*b*). Using the more-than-once model, we observed a significant repetition effect (table 1), while the order effect was not significant (table 1 and figure 3*c*). The non-significant order effect may indicate that when planning to use functions participants followed an injunctive rule and were less affected by the subjective value of using functions. This result may also be a consequence of not actually being engaged in the code writing, as such engagement could potentially update their perception of the subjective value.

### 3.3. Experiment 3

Finally, Experiment 3 was designed to evaluate whether planning combined with immediate implementation of the plan (i.e. combining Experiment 1 and 2) would yield a larger cost–benefit effect. As in Experiment 2, in each task in Experiment 3 participants were initially asked to make a planning decision. Immediately after each planning task they were then required to code the task, as in Experiment 1. Hence in this experiment, planning and coding were interwoven. The need to plan on the one hand while experiencing the cost of cognitive effort when programming the task on the other hand may serve to increase the effect of cost–benefit considerations.

**Figure 4.**
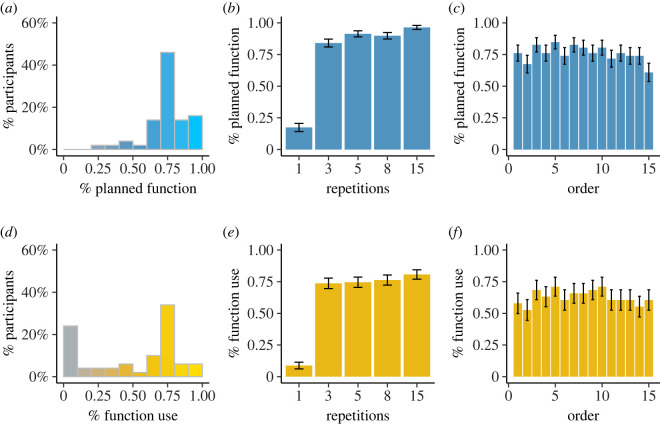
Computer-programming decisions in Experiment 3. (*a*) Percentage distribution of planning to code with functions in Experiment 3. The subset of participants who avoided functions at least once followed a more-than-once rule when planning their solution (*b*), and did not show any order effect when planning to use functions (*c*). Panels (*d–f*) show the same information as (*a–c*) for writing code with functions immediately after planning. The patterns of planning and coding were not sensitive to cost–benefit considerations.

In Experiment 3, we observed that all the participants planned to use functions at least once, and many of them planned to use functions in all tasks with more than one repetition (22 out of 50), a behaviour similar to that observed in Experiment 2, which included only the planning phase (figure 4*a*). This similarity was also confirmed using the Kolmogorov–Smirnov coefficient to examine the difference between the planning frequencies in Experiments 2 and 3, a difference that was far from significant (*D* = 0.095, *p* = 0.97).

Nevertheless, the participants' decision regarding how to write the code in Experiment 3 differed markedly from the parallel decision in Experiment 1 (figure 4*b*). In Experiment 3, we observed a lower function avoidance rate than in Experiment 1, as only 10 out of 50 participants avoided writing functions in all coding tasks in this experiment. In addition, 12 out of 50 participants wrote functions in all the tasks that included more than one repetition, in line with their decision in the planning phase. The resulting overall distribution of function use in Experiment 3 was significantly different than in Experiment 1 (*D* = 0.35, *p* = 0.0086). Note that the participants did not always comply with their planning decision in their coding, such that they did not always use functions in their code, even after stating they planned to do so.

We then examined the effects of repetition and order on planning and coding in Experiment 3, in the subset of participants who used functions at least once and avoided functions at least once (46 out of 50 participants). First, we found that the more-than-once model fitted the data better than the gradual repetition effect model, both for planning decisions to use functions (BIC gradual model: 564.11, BIC more-than-once model: 454.42, *χ*^2^ = 65.37) and for decisions to code with functions (BIC gradual model: 617.31, BIC more-than-once model: 470.72, *χ*^2^ = 101.2). It is worth mentioning that 'more-than-once' model represents a nonlinear relation, in which the benefits of functions rise sharply when the task includes repetitions. We used a formal model of nonlinear relationship, including a quadratic parameter, and found that it did not perform as well as the more-than-once model (a nonlinear model is included in electronic supplementary material, table ST4). In both cases, a significant repetition effect was observed (table 1 and figure 4*c* and *d*), while the order effect was not significant (table 1 and figure 4*e* and *f*). These results indicate that the effect of the planning phase on coding, as observed in the distribution of function use in Experiment 3, resulted in coding behaviour that followed an injunctive norm, similar to Experiment 2. This result indicates that the changes in coding behaviour were not mediated by a revised effort estimation process due to planning, but rather through use of the injunctive norm.

### 3.4. Task completion time

**Figure 5.**
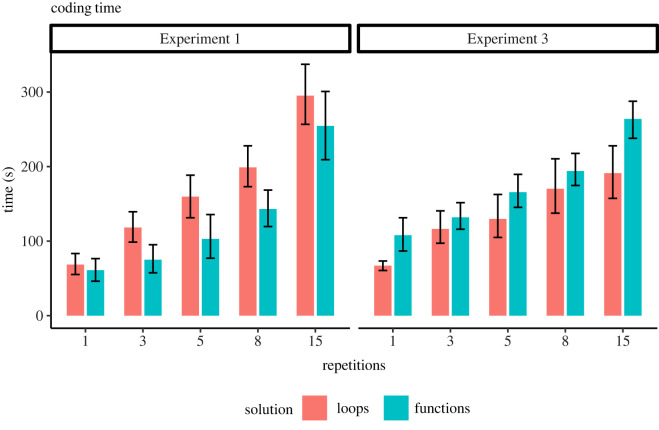
Solution times (seconds) for tasks with different number of repetitions, for functions and loops solutions, in Experiments 1 and 3. In Experiment 1, participants using functions solved the task faster than those using loops. The reverse is observed in Experiment 3. This analysis should be considered with caution, as participants were free to choose their preferred solution strategy.

Finally, changes in cost–benefit considerations may be manifested in the time it took participants to complete the different coding tasks. If functions provide benefit in that they speed up the coding of repetitive tasks, we can expect a faster completion time when functions are used. We, therefore, examined completion times in Experiments 1 and 3 (figure 5). The overall pattern indicates that in Experiment 1 participants using functions completed tasks faster than participants using loops. However, in Experiment 3 the pattern is opposite, with participants using functions spending more time than participants using loops, especially in the low repetition conditions. We conducted a mixed-effect linear regression, with completion time in seconds as dependent variable, and solution type (loops/functions) and repetitions within each task as independent variables. This analysis was carried out independently for each experiment. In Experiment 1 we found a significant main effect of repetitions (estimate ± s.e.: 51.74 ± 3.36, *t*_521.21_ = 15.41, *p* < 0.001), and a main effect of solution type (estimate ± s.e.: 46.03 ± 14.08, *t*_170.59_ = 3.27, *p* = 0.0013), as functions were generally faster than loops (full results in electronic supplementary material, table ST6). In Experiment 3 we found a significant main effect of repetitions (estimate ± s.e.: 35.12 ± 2.3, *t*_740.44_ = 12.88, *p* < 0.001), and a main effect of solution type (estimate ± s.e.: −38.28 ± 9.06, *t*_382.96_ = −4.22, *p* < 0.001), as functions were generally slower than loops (full results in electronic supplementary material, table ST6). This difference between Experiments may be related to the overuse of functions by participants in Experiment 3, indicating the cost of writing functions. However, this analysis should be considered with caution. We allowed participants to choose which solution to code (our main measurement), and therefore did not have within-participants comparison of solutions, and the separation for two solutions is biased (as the histograms of function use reveal). It may therefore be that participants that used the loops solution in Experiment 1 would have spent longer time if they opted for writing functions, and vice versa in Experiment 3. Therefore, it may not straightforwardly support a conclusion that writing functions is a faster solution than using loops for all participants.

## 4. Discussion

In this study, we set out to examine whether and how cost–benefit considerations affect the decision to exert cognitive effort in a computer-programming task. Specifically, we were interested in how such considerations fare against other factors that may affect such decisions. We manipulated the number of repetitions required for a computer-programming task based on the assumption that an effect of repetitions on decisions would indicate the role of cost–benefit considerations, while the lack of such an effect may stem either from indifference between the two coding strategies, or a decision process based on other factors, such as biased cognitive effort estimation and compliance with an injunctive norm. The results of our first experiment showed that less than half of the participants exhibited a cost–benefit decision pattern that was sensitive to the number of repetitions in a task. The rest of the participants (more than half) avoided writing reusable code altogether. In Experiments 2 and 3 we asked participants to plan how they would solve the task and to indicate their decision before solving the task. We found an overwhelming difference in the decisions made in these experiments compared with those made in Experiment 1 (coding only): most participants indicated they planned to use functions in all tasks that involved repetitions. Note that their decisions were not sensitive to the number of repetitions, provided there was more than one repetition. This indicates that their planning was not related to the preference observed in Experiment 1. Such pattern can arise from a nonlinear increase in cost of using loops, which will make functions more useful even with three repetitions. However, this pattern is also in line with a more-than-once injunctive norm, which may be triggered following the explicit planning question. These results indicate that the decisions made by programmers are flexible and relatively easy to manipulate when nudged by a planning activity, but are less affected by cost–benefit considerations.

Our results point to a new direction that should be considered in studying cognitive effort, and indeed other types of efforts and utility-based decision-making. The current approach in this field highlights cost–benefit considerations, which predict sensitivity to the amount of effort and the outcome of each option [1,2,23]. This approach builds on numerous experimental results that show a gradual increase in the likelihood of engaging in a cognitive effortful activity as the expected rewards increase, along with greater willingness to expend higher effort to gain greater rewards [4,7,10,39–41]. However, as demonstrated here, some other considerations and factors may affect this pattern, making behaviour more rigid and indifferent to changes in the cost and effort demands of the task at hand. We observed that many of our participants did not choose to write functions to solve the programming task, a strategy that is associated with a single high initial investment of cognitive effort but rather chose the clone-and-tweak strategy in which cognitive effort is initially low and increases with repetitions. This result may indicate an indifference toward the effort demands of the two coding strategies, with benefits of writing functions never outweighing the benefits of clone-and-tweak. However, the finding that at least some participants were more likely to use functions when the number of repetitions in the task increased (repetition effect), and the finding that some participants were more likely to use functions after completing some tasks (order effect), suggest that at least some of this effect is driven by biased effort-estimation. This bias may be due to a Pavlovian avoidance response to high investments or high costs [24]. Participants may have stopped planning beyond the initial costs of each option and were therefore biased in their effort estimation, leading to a rigid pattern of decisions. The cost–benefit framework should therefore incorporate the way in which cognitive effort is estimated as well as the notion that the process of estimation demands cognitive effort, especially when there is uncertainty concerning the amount of effort required in the future [5,25].

In Experiments 2 and 3, we observed rigid behaviour that was not sensitive to the number of repetitions but in a different way from the pattern observed in Experiment 1. This may be explained by a sudden change in cost–benefit estimation, under which the benefit of functions outweighs the cost of cognitive effort even in three repetitions. However, another explanation that is also related to the planning procedure introduced in Experiments 2 and 3, is that participants seemed to follow a more-than-once injunctive norm when prompted to state how they plan to solve the task. Adherence and conformity to social norms are known to have positive outcomes for the individual and may provide social motivation [42–47]. Normative considerations may completely offset cost–benefit calculations, as demonstrating one's willingness to pay a cost (cognitive effort) to adhere to a norm may serve as a strong social signal [48]. While one may argue that these external motivations do nothing more than add to the benefits associated with cognitive effort, we argue that this perspective may not be useful. Normative considerations may be associated with a variety of actions, regardless of their demand for cognitive effort, and therefore undermine the predictability of cost–benefit considerations. In addition, such normative effects are categorical and not sensitive to the amount of effort and its outcome. We therefore suggest that cost–benefit considerations may constitute only one factor among many others that determine the amount of cognitive effort to be exerted. In cases where normative rules apply, especially in social contexts, deontological and normative considerations may have a greater effect than cost–benefit considerations [49].

Our work demonstrates the value of applying theoretical predictions from psychology to problems in other fields of research and points to the mutual benefits of multidisciplinary research. How to understand the cognitive processes underlying programmers' decisions and how to promote efficient and reusable programming practices are prevalent issues in the field of software engineering [20–22,29,50]. For example, tools have been developed to detect cloning and replace it with reusable functions [50]. Our findings that programmers can be nudged to follow an injunctive norm—the more-than-once norm—and completely reverse their initial decision may be useful in developing real-world interventions. On the other hand, applying theory-based decisions in a new context that extends beyond the more traditional cognitive psychology tasks allowed us to examine how theoretical predictions fare in a more complex context, to delineate their limitations and to point to new research directions in the fields of decision-making, cognitive effort and motivation.

Our work has some limitations that should be acknowledged when interpreting the results and when designing future studies and applications. First, our participants were university students studying information systems. Such students may be more prone to follow the injunctive norms learned in class. Nevertheless, they still avoided using reusable code when not prompted by a planning instruction. The impact of such recommended best practices may diminish as they spend more time in professional environments, making our findings less readily applicable to experienced programmers or those who were not formally trained. In such cases, however, we suggest it may be useful to stress the normative aspects of programming and review the code and coding process, not only the outcome of the programming task, to promote the use of these best practices. An additional limitation of our current work is its focus on relatively simple computer-programming tasks, where the benefits of writing reusable code may not be immediately apparent. In more complicated tasks and work settings, code reuse has considerably greater benefits and may even emerge from the structure of the task. Nevertheless, research has found that even in these scenarios, code reuse is frequently lacking, causing frustration to team leaders and project managers [20,29]. In line with previous studies on cognitive effort, in our current study, we were able to examine the effect of experimental manipulation by using a simple scenario and therefore draw straightforward theoretical predictions.

To conclude, we examined how cost–benefit considerations affect the decision to exert cognitive effort in a real-world computer-programming task. We found that cost–benefit considerations had a limited effect on participants’ decisions, which were overwhelmingly determined by other factors that were less sensitive to changes in the costs and benefits of the different coding strategies. Future investigations of cognitive effort should strive to understand how different factors such as bias effort estimation and external motivations such as norms can be integrated into current theories of cognitive effort and decision-making. Inspiration from problems in other research fields may provide fertile ground for such theoretical insights and yield applicable solutions for real-world problems.

## Data Availability

Anonymized data and scripts used in this paper are available at https://osf.io/zd27r/?view_only=a26057c27cec4e1bbde276f76a1c2b4d. The data are provided in electronic supplementary material [51].

## References

[1] Shenhav A, Prater Fahey M, Grahek I. 2021 Decomposing the motivation to exert mental effort. Curr. Dir. Psychol. Sci. **30**, 307-314. (10.1177/09637214211009510) 34675454 PMC8528169

[2] Kool W, Botvinick M. 2018 Mental labour. Nat. Hum. Behav. **2**, 899-908. (10.1038/s41562-018-0401-9) 30988433

[3] Müller T, Apps MAJ. 2019 Motivational fatigue: a neurocognitive framework for the impact of effortful exertion on subsequent motivation. Neuropsychologia **123**, 141-151. (10.1016/j.neuropsychologia.2018.04.030) 29738794

[4] Vogel T, Savelson Z, Otto AR, Roy M. 2020 Forced choices reveal a trade-off between cognitive effort and physical pain. eLife **9**, e59410. (10.7554/eLife.59410) 33200988 PMC7714391

[5] Kool W, Gershman SJ, Cushman FA. 2018 Planning complexity registers as a cost in metacontrol. J. Cogn. Neurosci. **30**, 1391-1404. (10.1162/jocn_a_01263) 29668390

[6] Apps MAJ, Grima LL, Manohar S, Husain M. 2015 The role of cognitive effort in subjective reward devaluation and risky decision-making. Sci. Rep. **5**, 16880. (10.1038/srep16880) 26586084 PMC4653618

[7] Westbrook A, Kester D, Braver TS. 2013 What is the subjective cost of cognitive effort? Load, trait, and aging effects revealed by economic preference. PLoS ONE **8**, e68210. (10.1371/journal.pone.0068210) 23894295 PMC3718823

[8] Shenhav A, Musslick S, Lieder F, Kool W, Griffiths TL, Cohen JD, Botvinick MM. 2017 Toward a rational and mechanistic account of mental effort. Annu. Rev. Neurosci. **40**, 99-124. (10.1146/annurev-neuro-072116-031526) 28375769

[9] Yan X, Otto AR. 2020 Cognitive effort investment and opportunity costs in strategic decision-making: an individual differences examination. Pers. Individ. Dif. **167**, 110283. (10.1016/j.paid.2020.110283)

[10] Kool W, Botvinick M. 2014 A labor/leisure tradeoff in cognitive control. J. Exp. Psychol. Gen. **143**, 131-141. (10.1037/a0031048) 23230991 PMC3739999

[11] Hazzan O. 1999 Reducing abstraction level when learning abstract algebra concepts. Educ. Stud. Math. **40**, 71-90. (10.1023/A:1003780613628)

[12] Kramer J. 2007 Is abstraction the key to computing? Commun. ACM **50**, 36-42. (10.1145/1232743.1232745)

[13] Hazzan O. 2003 How students attempt to reduce abstraction in the learning of mathematics and in the learning of computer science. Comput. Sci. Educ. **13**, 95-122. (10.1076/csed.13.2.95.14202)

[14] Hazzan O, Kramer J. 2016 Assessing abstraction skills. Commun. ACM **59**, 43-45. (10.1145/2926712)

[15] Mow IC. 2012 Analyses of student programming errors in java programming courses. J. Emerg. Trends Comput. Inf. Sci. **3**, 739-749.

[16] Ciborowska A, Kraft NA, Damevski K. 2018 Detecting and characterizing developer behavior following opportunistic reuse of code snippets from the web. In Proc. of the 15th Int. Conf. on Mining Software Repositories, pp. 94-97. New York, NY: ACM.

[17] Basili VR, Briand LC, Melo WL. 1996 How reuse influences productivity in object-oriented systems. Commun. ACM **39**, 104-116. (10.1145/236156.236184)

[18] Hashim K, Key E. 1996 A software maintainability attributes model. Malaysian J. Comput. Sci. **9**, 92-97.

[19] Gershenson JK, Prasad GJ, Zhang Y. 2003 Product modularity: definitions and benefits. J. Eng. Des. **14**, 295-313. (10.1080/0954482031000091068)

[20] Reinhartz-Berger I, Tomer A, Grossman M. 2018 Reuse Considerations in Evolving Software Products: The Software Product Line Perspective. In *MODELS* *Workshops*, pp. 610-619. See http://ceur-ws.org/Vol-2245/me_paper_5.pdf.

[21] Fowler M. 2018 Refactoring: improving the design of existing code, 2nd edn. Boston, MA: Addison-Wesley Professional.

[22] Goodliffe P. 2007 Code craft: the practice of writing excellent code. San Francisco, CA: No Starch Press.

[23] Westbrook A, Braver TS. 2015 Cognitive effort: a neuroeconomic approach. Cogn. Affect. Behav. Neurosci. **15**, 395-415. (10.3758/s13415-015-0334-y) 25673005 PMC4445645

[24] Huys QJM, Eshel N, O'Nions E, Sheridan L, Dayan P, Roiser JP. 2012 Bonsai trees in your head: how the Pavlovian system sculpts goal-directed choices by pruning decision trees. PLoS Comput. Biol. **8**, e1002410. (10.1371/journal.pcbi.1002410) 22412360 PMC3297555

[25] Keramati M, Smittenaar P, Dolan RJ, Dayan P. 2016 Adaptive integration of habits into depth-limited planning defines a habitual-goal–directed spectrum. Proc. Natl Acad. Sci. USA **113**, 12 868-12 873. (10.1073/pnas.1609094113) 27791110 PMC5111694

[26] Huys QJM, Cools R, Gölzer M, Friedel E, Heinz A, Dolan RJ, Dayan P. 2011 Disentangling the roles of approach, activation and valence in instrumental and Pavlovian responding. PLoS Comput. Biol. **7**, e1002028. (10.1371/journal.pcbi.1002028) 21556131 PMC3080848

[27] Huys QJM, Lally N, Faulkner P, Eshel N, Seifritz E, Gershman SJ, Dayan P, Roiser JP. 2015 Interplay of approximate planning strategies. Proc. Natl Acad. Sci. USA **112**, 3098-3103. (10.1073/pnas.1414219112) 25675480 PMC4364207

[28] Omar A, Hadar I, Leron U. 2017 Investigating the under-usage of code decomposition and reuse among high school students: the case of functions. Lect. Notes Bus. Inf. Process. **286**, 92-98. (10.1007/978-3-319-60048-2_9)

[29] Mann ZA. 2006 Three public enemies: cut, copy, and paste. Computer (Long Beach Calif.). **39**, 31-35. (10.1109/MC.2006.246)

[30] White KM, Smith JR, Terry DJ, Greenslade JH, McKimmie BM. 2009 Social influence in the theory of planned behaviour: the role of descriptive, injunctive, and in-group norms. Br. J. Soc. Psychol. **48**, 135-158. (10.1348/014466608X295207) 18435863

[31] Lapinski MK, Rimal RN. 2005 An explication of social norms. Commun. Theory **15**, 127-147. (10.1093/ct/15.2.127)

[32] Bear A, Knobe J. 2017 Normality: part descriptive, part prescriptive. Cognition **167**, 25-37. (10.1016/j.cognition.2016.10.024) 27842702

[33] Borsari B, Carey KB. 2003 Descriptive and injunctive norms in college drinking: a meta-analytic integration. J. Stud. Alcohol **64**, 331-341. (10.15288/jsa.2003.64.331) 12817821 PMC2431131

[34] Bear A, Bensinger S, Jara-Ettinger J, Knobe J, Cushman F. 2020 What comes to mind? Cognition **194**, 104057. (10.1016/j.cognition.2019.104057) 31505322

[35] Gelman A, Hill J. 2006 Data analysis using regression and multilevel/hierarchical models. Cambridge, UK: Cambridge University Press.

[36] West BT, Welch KB, Galecki AT. 2014 Linear mixed models: a practical guide using statistical software. Boca Raton, FL: CRC Press.

[37] Kuznetsova A, Brockhoff PB, Christensen RHB. 2017 lmerTest Package: tests in linear mixed effects models. J. Stat. Softw. **82**, 1-26. (10.18637/jss.v082.i13)

[38] Otto AR, Braem S, Silvetti M, Vassena E. 2021 Learning the marginal value of mental effort over time. *PsyArXiv*. (10.31234/osf.io/9np6f) 35389742

[39] Lockwood PL, Hamonet M, Zhang SH, Ratnavel A, Salmony FU, Husain M, Apps MAJ. 2017 Prosocial apathy for helping others when effort is required. Nat. Hum. Behav. **1**, 1-10. (10.1038/s41562-017-0131) PMC5555390 28819649

[40] Contreras-Huerta LS, Pisauro MA, Apps MAJ. 2020 Effort shapes social cognition and behaviour: a neuro-cognitive framework. Neurosci. Biobehav. Rev. **118**, 426-439. (10.1016/j.neubiorev.2020.08.003) 32818580

[41] Frömer R, Lin H, Dean Wolf CK, Inzlicht M, Shenhav A. 2021 Expectations of reward and efficacy guide cognitive control allocation. Nat. Commun. **12**, 1030. (10.1038/s41467-021-21315-z) 33589626 PMC7884731

[42] Toelch U, Dolan RJ. 2015 Informational and normative influences in conformity from a neurocomputational perspective. Trends Cogn. Sci. **19**, 579-589. (10.1016/j.tics.2015.07.007) 26412096

[43] Klucharev V, Hytönen K, Rijpkema M, Smidts A, Fernández G. 2009 Reinforcement learning signal predicts social conformity. Neuron **61**, 140-151. (10.1016/j.neuron.2008.11.027) 19146819

[44] Hertz U. 2021 Learning how to behave: cognitive learning processes account for asymmetries in adaptation to social norms. Proc. R. Soc. B **288**, 20210293. (10.1098/rspb.2021.0293) PMC8170188 34074119

[45] Elster J. 1989 The cement of society: a survey of social order. Cambridge, UK: Cambridge University Press.

[46] Fehr E, Schurtenberger I. 2018 Normative foundations of human cooperation. Nat. Hum. Behav. **2**, 458-468. (10.1038/s41562-018-0385-5) 31097815

[47] Yoeli E, Hoffman M, Rand DG, Nowak MA. 2013 Powering up with indirect reciprocity in a large-scale field experiment. Proc. Natl Acad. Sci. USA **110**, 10 424-10 429. (10.1073/pnas.1301210110) PMC3690615 23754399

[48] Jordan JJ, Hoffman M, Bloom P, Rand DG. 2016 Third-party punishment as a costly signal of trustworthiness. Nature **530**, 473-476. (10.1038/nature16981) 26911783

[49] Everett JAC, Pizarro DA, Crockett MJ. 2016 Inference of trustworthiness from intuitive moral judgments. J. Exp. Psychol. Gen. **145**, 772-787. (10.1037/xge0000165) 27054685

[50] Jablonski P, Hou D. 2007 CReN: a tool for tracking copy-and-paste code clones and renaming identifiers consistently in the IDE. In Proc. of the 2007 OOPSLA Workshop on Eclipse Technology EXchange, pp. 16-20. New York, NY: Association for Computing Machinery.

[51] Lachman I, Hadar I, Hertz U. 2022 Cost-benefit considerations have limited effect on the decision to exert cognitive effort in real-world computer-programming tasks. *Figshare*. (10.6084/m9.figshare.c.6035787) PMC9214280 35754993

